# Practice and knowledge about diagnosis and treatment of alpha-1 antitrypsin deficiency in Spain and Portugal

**DOI:** 10.1186/s12890-016-0222-4

**Published:** 2016-04-27

**Authors:** Cristina Esquinas, Miriam Barrecheguren, Maria Sucena, Esther Rodriguez, Sandra Fernandez, Marc Miravitlles

**Affiliations:** Pneumology Department, Hospital Universitari Vall d’Hebron, CIBER de Enfermedades Respiratorias (CIBERES), Barcelona, Spain; Pneumology Department, Centro Hospitalar de São João, Porto, Portugal; Scientific and Medical Affairs, Grifols, Barcelona, Spain

**Keywords:** Clinical practice, Knowledge, Alpha-1 antitrypsin deficiency, Management

## Abstract

**Background:**

Determining physicians’ awareness about alpha-1 antitrypsin (AAT) deficiency (AATD) may help to explain the discrepancy between the observed and expected number of patients diagnosed with this disease.

This study was designed to assess the opinions on knowledge, practice pattern and attitude regarding AATD among physicians in Spain and Portugal.

**Methods:**

An online anonymous survey was performed on pulmonologists (*n* = 100), internal medicine specialists (IMS) (*n* = 100) and primary care physicians (PCP) (*n* = 176). Of the total number of physicians, 221 were from Spain, and 155 were from Portugal. Physicians answered 21 questions related to their personal and professional profile, knowledge regarding AATD and chronic obstructive pulmonary disease (COPD), performance and attitude about AATD, and use of augmentation therapy. Responses were ranked on a 4-point scale indicating the level of agreement. In addition, some of the responses were rated as either “low” or “high” indicating the level of knowledge the respondent felt he/she possessed.

**Results:**

Only 14 % of physicians reported to “know very well” about AATD (3.3 [SD 0.6] for pulmonologists vs. 2.64 [SD 0.60] for IMS and 2.48 [SD 0.71] for PCP; *p* < 0.001). Only 45.2 % of physicians correctly answered “<50 mg/dL” as the threshold value of serum AAT to be considered severe AATD (55.0 % of pulmonologists vs. 47.0 % of IMS and 38.6 % of PCP; *p* = 0.001). Choice of the correct answer did not agree with those physicians self-declaring a high level of AATD knowledge (51.2 %). A total of 43.9 % of physicians correctly identified all diseases or conditions in a list associated or not with AATD. A similar trend was detected when identifying which conditions would be responsive to augmentation therapy (<50 %). Only 15.8 % of specialists performed AATD testing in all patients with COPD (27.0 % pulmonologists, 12.6 % PCP; *p* = 0.001).

**Conclusion:**

The results suggest that a knowledge gap may be contributing to the underdiagnosis of AATD. Physicians in Spain and Portugal showed a marked lack of awareness of their shortcomings in knowledge about AATD, and in general did not follow guidelines and recommendations for AATD testing.

## Background

Alpha-1 antitrypsin (AAT) deficiency (AATD) is a common but underdiagnosed human hereditary disorder characterized by impaired or defective production of AAT protein in the liver [1], which mainly results in compromised pulmonary protection. In AATD, protease inhibitor (PI)-deficient alleles (primarily PI*Z and PI*S) are inherited from the AAT gene locus instead of the normal allele (PI*M). In Europe, there are differences in gene frequencies between geographic regions [2] and the PI*S allele is more common than the PI*Z allele in western countries, such as Spain and Portugal [3].

Balanced protease-antiprotease function is maintained mainly by AAT in the healthy lung through inhibition of human neutrophil elastase [4, 5], an enzyme that degrades basement membrane components of lung epithelium and connective tissue, activates other proteinase proenzymes, and is chemoattractant for inflammation cells. AAT excess can lead to destruction of alveolar walls [6] and is associated with increased risk of early-onset pulmonary emphysema as well as liver disease due to accumulation of misfolded protein in hepatocytes [7].

In addition to liver disease, panniculitis and vasculitis also have been associated with AATD. Adult-onset AATD-associated liver disease manifests as cirrhosis and fibrosis [8]. Panniculitis occurs in approximately 1 of 1000 individuals with AATD [9, 10]. AATD is associated with the risk of C-ANCA-positive vasculitis, such as polyangiitis with granulomatosis [11].

Normal AAT concentration measured by nephelometry in serum is 120–220 mg/dL [12]. Guidelines for the diagnosis and treatment of AATD determine the protective threshold level of serum AAT at 50 mg/mL [10–14]. Recommendations and guidelines of healthcare institutions, such as the World Health Organization, The Spanish Society of Pneumology and Thoracic Surgery (SEPAR) and the American and European Thoracic/Respiratory Societies (ATS/ERS), indicate that all chronic obstructive pulmonary disease (COPD) subjects and adults with nonreversible asthma should be tested for AATD at least once during their lifetime [10, 14, 15]. In that regard, the Spanish Registry of Patients with AATD (REDAAT) has developed a AATD free-cost detection program using dried blood spots that is available for physicians.

However, even with the availability of AATD screening and detection programs [16–23], the number of patients diagnosed with AATD is much less than expected according to epidemiologic studies [24, 25], which suggests that adherence to recommendations is lacking. Understanding the level of knowledge physicians have about all aspects of AATD is crucial to determining the possible reasons why such a delay in the diagnosis of AATD still exists [26].

This study explored the awareness of physicians in Spain and Portugal towards the diagnosis of AATD. The possible relationship between the results of the survey and the underdiagnosis of AATD is discussed.

## Methods

### Study design

This cross-sectional, observational study was conducted in July and August of 2014 by means of an online survey. This study did not require institutional protocol approvals or special consents because the nature of the survey was anonymous. The target physician population consisted of pulmonologists, internal medicine specialists (IMS) and primary care physicians PCP) in Spain and Portugal who treat at least five patients with COPD per month. A total of 4714 physicians (2700 from Spain and 1414 from Portugal) were invited to participate in the survey (link accessed) who were randomly identified from an international medical marketing research database according to geographic distribution and proportion of each specialty. Those who accepted the invitation and responded to the survey were verified to meet the study requirements and de-identified to guarantee anonymity.

### Study objectives

The main objective of this study was to assess the level of knowledge and practice pattern regarding AATD among physicians in Spain and Portugal. Secondary objectives were to explore their practice pattern and attitude regarding AATD based on clinical specialty (pulmonologists, IMS and PCP).

### Characteristics of the questionnaire and survey data-handling

Physicians answered a 15-minute, 21–question survey about their personal and professional profile (e.g., age, sex, location, specialty, experience) and their knowledge regarding AATD and COPD (e.g., threshold value of serum AAT, diseases or conditions associated with AATD, criteria for the indication of AAT augmentation therapy). The survey also collected information on physician’s practice pattern and attitude about AATD (e.g., testing practice, communication with patient and laboratory) and their use of augmentation therapy. Responses were rated according to a numerical Likert scale, where “none at all”/“totally disagree” = 1, “a little”/“somewhat disagree” = 2, “average”/“somewhat agree” = 3, and “very well”/“totally agree” = 4. Some responses to knowledge-based questions (e.g, “How much you think that you know about AATD?”) were rated in 2 categories of knowledge for secondary analysis (low level: to know “none at all” or “a little”; high level: to know “average” or “very well”). This questionnaire was similar to a previously published survey performed on German and Italian physicians [26] with the addition of a few questions related to knowledge and opinions about AATD. The survey data were analyzed based on the country in which the physician practiced and/or his/her clinical specialty. The questionnaire is available as an appendix to this report.

### Statistical analysis

Quantitative variables are shown as mean and standard deviation (SD) or median and interquartile range (IQR). In the case of qualitative variables, frequency and percentages were determined. Differences between specialties and country were determined using chi-square test (or Fisher’s test when the expected frequencies <5) for qualitative variables, and by ANOVA test or T Student test for quantitative variables. In all cases, statistically significant differences were considered *p* < 0.05. Statistical analysis was performed using the SPSS 19.0 Software (Chicago, IL, USA).

## Results

### Physician profile

A total of 376 physicians participated in the study, 221 (58.8 %) from Spain and 155 (41.2 %) from Portugal. Survey participants included 100 pulmonologists (26.6 %; 60 in Spain and 40 in Portugal), 176 PCP (46.8 %; 101 in Spain and 75 in Portugal) and 100 IMS (26.6 %; 60 in Spain and 40 in Portugal). Mean age of the surveyed physicians was 46.7 (SD 9.6) years, and 63 % were male. Physicians represented a mean professional experience of 18.7 (SD 8.9) years and treated a median of 40 (IQR = 20–90) patients with COPD per month. No differences were observed between countries in these variables (data not shown).

### Self-declared knowledge and opinions about AATD and other respiratory diseases

**Fig. 1:**
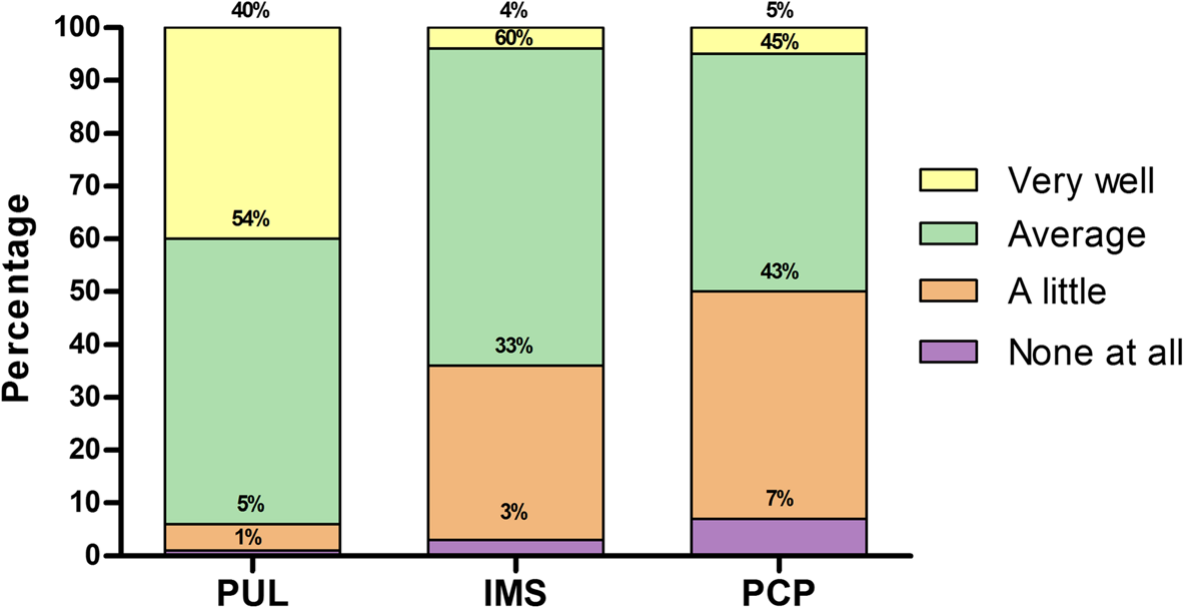
Self-reported level of knowledge about alpha-1 antitrypsin deficiency (*p* < 0.001 among groups) (PUL: pulmonologists, PCP: primary care physicians, IMS: internal medicine specialists)

The level of knowledge regarding AATD was lower compared with COPD on the 4-point scale (2.75 [SD 0.75] vs. 3.7 [SD 0.5], respectively; *p* = 0.001). Only 14 % of physicians reported to know “very well” about AATD. Pulmonologists (3.3 [SD 0.6]) declared to have a higher level of knowledge in AATD than IMS (2.64 [SD 0.60]) and PCP (2.48 [SD 0.71]) (*p* < 0.001). Figure 1 shows the degree of knowledge by percentages using the Likert scale according to specialists. For COPD, 65.2 % of physicians declared to know “very well”. In particular, pulmonologists were the specialists who declared to better know about COPD (3.86 [SD 0.38] for pulmonologists vs. 3.73 [SD 0.45] for IMS and 3.48 [SD 0.50] for PCP; *p* < 0.001).

The main source of information for AAT deficiency was textbooks (79.7 %), followed by scientific articles (73.5 %). Other sources of information used by almost half of the physicians were clinical guidelines (46.8 %), scientific congresses (45.4 %) and advice from experts and colleagues (43.7 %).

Knowledge about AATD was evaluated by three questions. In the first question, only 45.2 % of physicians correctly answered “<50 mg/dL” as the threshold value of serum AAT to be considered severe AATD. Pulmonologists had the highest rate of choosing the correct answer compared to physicians from other specialties (55.0 % for pulmonologists vs. 47.0 % of IMS and 38.6 % of PCP; *p* = 0.001). When these results were compared against the self-declared knowledge about AATD, only 51.2 % of those who declared to know “average” or “very well” about AATD correctly chose “<50 mg/dL.” This percentage was higher than that observed in those who declared to know “a little” or “none at all” (33.8 %; *p* = 0.001).

In the second question, physicians were asked to pick from a list of 6 diseases or conditions which ones can be associated with AATD. “Hepatic cirrhosis” was correctly chosen by 72.8 % (PCP), 73 % (IMS) and 93 % (pulmonologists) of physicians as being a disease associated with AATD, while less than 44 % of all physicians correctly identified “panniculitis” and “vasculitis”. Diseases not associated with AATD, such as “heart disease” and “arterial hypertension” were selected by 8.5 and 19.9 % of physicians, respectively. A total of 43.9 % of physicians identified the three correct answers and the two incorrect answers, with no statistically significant differences among specialties (46.0 % of pulmonologists, 49.0 % of IMS and 39.8 % of PCP, *p* = 0.35). No differences were observed when comparisons were made with respect to the degree of self-declared knowledge (*p* = 0.670).

The third question about AATD knowledge referred to the criteria for the indication of AAT augmentation therapy. Only 18 physicians (4.8 %) identified the correct answers (“older than 18 years of age,” “severe AATD,” “emphysema,” “FEV1 36–60 %,” and “not active smoker”) and identified “liver disease” as the incorrect answer. No differences were observed regarding the degree of self-declared knowledge of AATD (*p* = 0.102). Physicians in Portugal showed a lower level of knowledge about criteria for augmentation therapy than did physicians in Spain. Detailed information according to specialty and country is shown in Table 1.

**Table 1:** Responses to the question about criteria for the indication of augmentation therapy for alpha-1 antitrypsin deficiency (n, %)

	Spain	Portugal	*P* value	PUL	IMS	PCP	*P* value	Total
	*N* = 221	*N* = 155		*N* = 100	*N* = 100	*N* = 176		*N* = 376
Severe AATD	190 (86.0)	103 (66.5)	<0.001	96 (96.0)	77 (77.0)	120 (68.2)	<0.001	293 (77.9.0)
Emphysema	146 (66.1)	69 (44.5)	0.001	71 (71.0)	55 (55.0)	89 (50.6)	0.004	215 (57.2)
FEV1	99 (44.0)	54 (34.8)	NS	63 (63.0)	36 (36.0)	54 (30.7)	<0.001	153 (40.7)
-<=35 %	28 (12.7)	17 (11.0)	NS	20 (20.0)	12 (12.0)	13 (7.4)	0.008	45 (12.0)
-36–60 %	71 (32.1)	25 (16.1)	0.001	42 (42.0)	18 (18.0)	36 (20.6)	<0.001	96 (25.5)
-61–80 %	7 (7.7)	16 (10.3)	NS	17 (17.0)	8 (8.0)	8 (4.5)	<0.001	33 (8.7)
Non smoker	99 (44.8)	56 (36.1)	NS	72 (72.0)	33 (33.0)	50 (28.1)	<0.001	155 (41.2)
Liver disease	80 (36.2)	42 (27.1)	NS	33 (33.0)	40 (40.0)	49 (27.1)	NS	122 (32.4)
Age >18 years	65 (29.4)	46 (29.7)	NS	46 (46.0)	23 (23.0)	42 (23.9)	<0.001	111 (29.5)

*PUL* pulmonologists, *IMS* internal medicine specialists, *PCP* primary care physicians, *AATD* Alpha-1 antitrypsin deficiency, *NS* nonsignificant

**Fig. 2:**
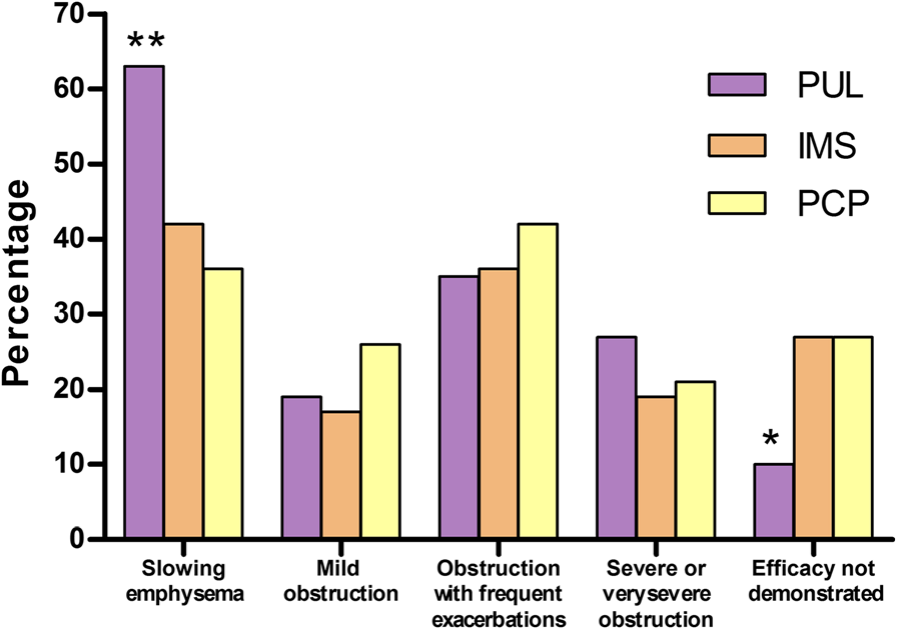
Conditions in which augmentation therapy with alpha-1 antitrypsin is effective according to physicians (*P = 0.001; **P = 0.002) (PUL: pulmonologists, PCP: primary care physicians, IMS: internal medicine specialists)

When physicians were asked about their opinion on the efficacy of AAT augmentation therapy for severe AATD, 44.9 % believed that therapy “slows the emphysema progression,” and there were differences among specialists (63.0 % of pulmonologists vs. 42.0 % of IMS and 36.4 % of PCP; *p* = 0.001). Lower percentages were observed for other settings Fig. 2.

### Routine AATD testing performance and management

**Fig. 3:**
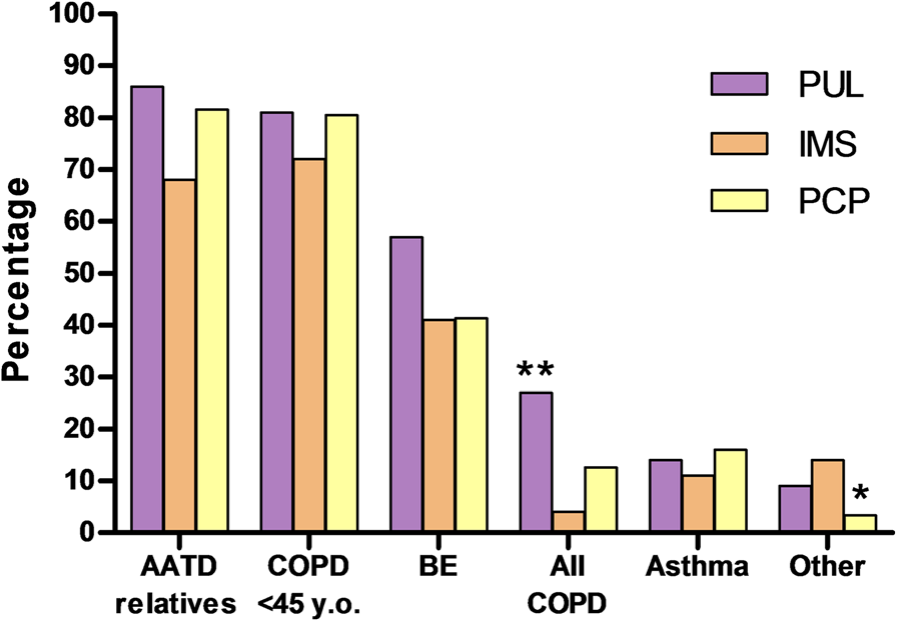
Clinical profile of patients tested by physicians for alpha-1 antitrypsin deficiency (*P = 0.010; **P = 0.001) (PUL: pulmonologists, PCP: primary care physicians, IMS: internal medicine specialists; AATD: Alpha-1 antitrypsin deficiency; COPD: Chronic Obstructive Pulmonary Disease; BE: bronchiectasis)

More than 80 % of physicians declared that they test family members of AATD patients and young COPD patients (<45 years of age) (Fig. 3). However, only 15.8 % of physicians commonly tested for AATD in all patients with COPD (27.0 % of pulmonologists, 12.6 % of PCP and 4 % of IMS; *p* = 0.001). Physicians with a high degree of self-declared knowledge performed AATD testing in all COPD patients more frequently than physicians with a lower degree (*p* < 0.05). The most common number of monthly requests for AATD was between 1 and 5 tests (73.6 % of physicians), regardless of specialty. Characteristics in frequency and number of tests by country are shown in Table 2. Physicians who reported not performing testing for AAT deficiency regularly cited referrals to another specialist (39.9 %) and the high cost of testing (39.0 %) as the main reasons for not testing, with some significant differences among specialties and country, as shown in Table 3. Up to 31.9 % of all physicians declared to have never treated any AATD patient with augmentation therapy.

**Table 2:** Diagnosis and management of alpha-1 antitrypsin deficiency by country (n, %)

	Spain	Portugal	Total sample
Frequency of request AATD testing	*N* = 221	*N* = 155	*N* = 376
Yes, regularly	51 (23.1)	17 (11)	68 (18.1)
Yes, occasionally	118 (53.4)	79 (51)	197 (52.4)
No	52 (23.5)	59 (38.1)	111 (29.5)
Number of test per month	*N* = 169	*N* = 96	*N* = 265
From physicians currently testing			
None	12 (7.1)	17 (17.7)	29 (10.9)
1–5	121 (71.6)	74 (77.1)	195 (73.6)
6–10	29 (17.2)	4 (4.2)	33 (12.5)
>10	7 (4.1)	1 (1)	8 (3)
Profile of patients tested	*N* = 169	*N* = 96	*N* = 265
From physicians currently testing			
All patients with COPD	30 (17.8)	12 (12.5)	42 (15.8)
COPD <45 years	140 (82.8)	83 (86.5)	223 (84.2)
Relatives AATD patients	144 (85.2)	81 (84,4)	225 (84.9)
Asthma	25 (14.8)	14 (14.1)	39 (14.7)
Bronchiectasis	89 (52.7)	45 (46.9)	134 (50.6)
Other conditions	13 (7.7)	13 (13.5)	26 (9.8)

*COPD* chronic obstructive pulmonary disease, *AATD* alpha-1 antitrypsin deficiency

**Table 3:** Physician’s reasons not to perform alpha-1 antitrypsin deficiency test. Data show totally agreement in each question (n, %)

	Spain	Portugal	*P* value	PUL	IMS	PCP	*P* value	Total
	*N* = 170	*N* = 138		*N* = 55	*N* = 91	*N* = 162		*N* = 308
Not available treatment	11 (6.5)	13 (9.4)	NS	6 (10.9)	10 (11.0)	8 (4.9)	NS	24 (7.8)
Not having time	20 (11.8)	11 (8.0)	NS	9 (16.0)	4 (4.0)	18 (11.0)	0.016	31 (10.1)
High economic cost	77 (45.3)	43 (31.2)	0.011	22 (40.0)	45 (49.5)	53 (32.7)	0.032	120 (39.0)
Too long to receive results	27 (15.9)	10 (7.2)	0.020	12 (21.8)	11 (12.0)	14 (8.6)	0.034	37 (12.0)
Not visiting any patient with AATD	10 (5.9)	39 (28.3)	<0.001	5 (9.0)	17 (18.0)	27 (16.7)	NS	49 (15.9)
Referred to other specialist	59 (34.7)	64 (46.4)	0.038	11 (20.0)	28 (30.0)	84 (52.0)	0.001	123 (39.9)
Any positive determination	23 (13.5)	2 (1.4)	<0.001	6 (10.0)	11 (12.0)	8 (5.0)	NS	25 (8.1)
Unclear interpretation of results	14 (8.2)	9 (6.5)	NS	4 (7.0)	6 (6.0)	13 (8.0)	NS	23 (7.5)
Other	31 (18.2)	25 (18.1)	NS	14 (25.5)	18 (19.0)	24 (14.8)	NS	56 (18.2)

*PUL* pulmonologists, *IMS* internal medicine specialists, *PCP* primary care physicians, *AATD* alpha-1 antitrypsin deficiency, *NS* nonsignificant

### Physician’s attitude and communication

Among the 265 physicians (70.5 %) who declared to regularly or occasionally test for AATD, 45.2 % totally agreed with the usefulness of the information provided by the laboratory, while 27.0 % totally agreed that the laboratory provided educational material about the implication of the test results for the patient. Pulmonologists were those most satisfied with the clarity of communication with the laboratory (57.7 %) compared to IMS and PCP who were less satisfied (32.1 and 25.2 %, respectively; *p* = 0.001).

Regarding communication with patient, only 16.6 % of all physicians clearly affirmed that they had educational materials about AATD available for patients and families. While 71.1 % of pulmonologists “totally agreed” with the clarity of communication with the patient, only 44.4 % of PCP did (*p* = 0.001). In addition, 12.5 % of physicians “totally agreed” with feeling uneasiness due to the genetic nature of the disease, and 11.3 % felt unsure about giving advices on prognosis, life style and treatments.

## Discussion

The results of this study performed by means of an online survey on physicians in Spain and Portugal indicate not only a poor knowledge about AATD in this target group, even in lung specialists, but also a pronounced lack of awareness of such shortcomings. It is possible that such knowledge gap contributes to the low rate of AAT testing done by physicians, particularly PCP. AATD testing is recommended for all COPD patients regardless of age or smoking history. Discrepancy between the observed and expected number of patients diagnosed with AATD has been repeatedly reported [16, 18, 24]. Insights on the physicians’ awareness, knowledge, understanding, attitude and practice pattern in AATD may provide a hint to explain the delay in the diagnosis of AATD.

As expected, pulmonologists were the specialists who mostly self-declared to know “very well” about both AATD and COPD the most, followed by IMS and PCP. However, the knowledge declared about AATD was significantly lower than COPD. This profile is in agreement with that reported by Greulich and coworkers for a similar study conducted in Germany and Italy [26]. When all of the physicians were considered, only 14 % declared to know “very well” about AATD. This value seems low, although it is difficult to determine the percentage level that one would consider such knowledge as acceptable.

Surprisingly, only 51.2 % of all physicians who self-declared to know “very well” about AATD identified correctly the “50 mg AAT/dL” in serum as the upper limit for severe AATD. This percentage was similar when only pulmonologists (55.0 %) and IMS (47.0 %) were considered. Similarly, less than half of all physicians were able to correctly identify in a list the diseases or conditions that can be associated with AATD and those that are not. Even lower knowledge was observed when less than 5 % of all physicians identified all the correct and incorrect criteria for the indication of AAT augmentation therapy. These results confirm that there is a low degree of knowledge about AATD in the physician specialties surveyed, even among those pulmonologists who believed to know “a lot” about AATD. Thus, AATD may be overlooked by physicians, particularly PCP, who declared even less knowledge about AATD, in accordance with other survey studies [26, 27].

In addition to the insufficient knowledge about AATD, and probably related to it, not all AAT determinations that would be desirable were performed. Despite recommendations and guidelines of healthcare institutions [10–15], only 4 % (IMS) to 27 % (pulmonologists) of physicians in Spain and Portugal performed AATD testing in all patients with COPD, although many tested young COPD patients and relatives of AATD patients. Reasons cited for not testing patients as well as percentages observed were similar to those described in Germany and Italy [26].

Since primary care is the central form of health access for patients with COPD, there is concern on how to engage PCP to reduce the underdiagnosis of AATD [28]. In our study, PCP felt the least comfortable with communicating to both the analysis laboratory and the patient.

In Spain, strategies such a detection program of AATD in COPD patients using dried blood spot have been performed. Results showed a feasible program at a reasonable cost per case detected (17). Pilot studies, such as the IDDEA web tool, aimed to coordinate the flow of data between the PCP, the analysis laboratory, and the pulmonologist have been developed for the screening and detection of AATD in the primary care setting [23]. Educational materials were also provided to the PCP to increase their awareness about AAT. However, our results suggest that before providing adequate diagnostic tools to physicians, particularly family doctors, there is still significant room for improvement in physician education and knowledge related to AATD. Further strategies such as inclusion of AAT testing in COPD guidelines, national screening programs with simpler testing approaches and an increase in physician education about AATD should help to improve in the AATD diagnosis.

Physician population surveyed in this study was considered balanced in account of the socio-demographic weight of the represented countries, practice setting and specialty. Although Spain and Portugal can be deemed more socio-culturally similar than Italy and Germany was in the study by Greulich and coworkers [26], some differences were found out. Explanation may lie on facts such as the unavailability of specific guidelines and a reference laboratory in Portugal. Although Portuguese professionals may use the ATS/ERS [10] or even the Spanish guidelines [13, 14] if necessary, that may represent a serious limitation.

Shortcomings and limitations typically associated with observational studies can affect this survey-based assessment performed on physicians in Spain and Portugal. Moreover, the survey was anonymous, gathered only the profile of those who accepted to participate, and focused on opinions and declarations about their own practice and self-declared knowledge about diagnosis and treatment of AATD, rather than based in daily routine quantifications. It is known that self-evaluation can be subjected to bias [29]. In fact, our results would be in agreement with the tendency to overestimate one’s knowledge and abilities.

## Conclusions

We found insufficient knowledge and unsatisfactory practice about AATD of physicians at all levels, from PCP to IMS and pulmonologists, which may be linked to insufficient adherence of the guidelines and recommendations for testing all COPD patients. The results of this study are consistent with the generalized underscreening and underdiagnosis of AATD.

## Ethics approval and consent to participate

This research did not involve patients or patients’ information; therefore the study did not fall under the Medical Research Involving Human Subjects Act and therefore did not require medical ethical approval.

## Consent for publication

“Not applicable”

## Availability of data and materials

Data are available upon request.

## Abbreviations

AATD: alpha-1 antitrypsin deficiency
ATS: American Thoracic Society
COPD: chronic obstructive pulmonary disease
ERS: European Respiratory Society
IMS: internal medicine specialists
IQR: interquartile range
PCP: primary care physicians
REDAAT: Spanish registry of patients with AATD
SD: standard deviation
SEPAR: Spanish Society of pneumology and thoracic surgery
